# Preliminary Evidence That Fiji Water Has Protective Effects against Aluminum Toxicity in Honey Bees (*Apis mellifera*)

**DOI:** 10.3390/insects14020211

**Published:** 2023-02-20

**Authors:** Kiri Li N. Stauch, Ana M. Chicas-Mosier, Charles I. Abramson

**Affiliations:** 1 Laboratory of Behavioral Biology and Comparative Psychology, Department of Psychology, Oklahoma State University, Stillwater, OK 66047, USA; 2 Center for Environmentally Beneficial Catalysis, University of Kansas, Lawrence, KS 66045, USA

**Keywords:** aluminum chloride, *Apis mellifera*, honey bees, silica

## Abstract

**Simple Summary:**

The honey bee (*Apis mellifera*) plays a crucial role as a pollinator in agricultural production. As such, it is important to protect the health of honey bees and their hives. One danger to honey bees is bioavailable aluminum chloride (AlCl3), which negatively impacts bee and hive health. Additionally, AlCl3 has been shown to cause impairments in foraging behavior and locomotion. We tested whether Fiji water reduced the impact of AlCl3 on honey bee rhythmicity, average daily activity, and mortality. The bees that received Fiji water and aluminum exhibited significant differences in rhythmicity, average daily activity, and mortality compared to the bees that received deionized (DI) water and aluminum. Overall, our findings provide tentative evidence that Fiji water may act as a protectant against the deleterious effects of AlCl3.

**Abstract:**

Researchers have determined that bioavailable aluminum chloride (AlCl3) may affect honey bee behavior (e.g., foraging patterns and locomotion) and physiology (e.g., abdominal spasms). The purpose of these experiments was to determine if Fiji water reduces the impacts of AlCl3 toxicity in bees by measuring circadian rhythmicity (number of times bees crossed the centerline during the day and night), average daily activity (average number of times bees crossed the centerline per day), and mortality rates (average number of days survived) using an automated monitor apparatus. Overall, the AlCl3 before and after Fiji groups had significantly higher average daily activity and rhythmicity rates compared to their respective AlCl3 before and after deionized water (DI) groups. One of the AlCl3 before DI groups exhibited no difference in rhythmicity rates compared to its respective AlCl3 after Fiji group. Overall, these results suggest that Fiji water might exert protective effects against AlCl3. The AlCl3 groups paired with Fiji water had higher activity and rhythmicity levels compared to the AlCl3 groups paired with DI. It is important for researchers to continue to study aluminum and possible preventatives for aluminum uptake.

## 1. Introduction

In 2017, the honey industry, which is comprised of production, importation, and packaging, contributed $4.7 billion to the United States economy and 147.6 million pounds of honey [1]. In addition to this economic boon, honey bee [*Apis mellifera* L. (Hymenoptera: Apidae)] pollination accounts for tens of billions of dollars in increased crop values every year [2]. As a result of this pollination dependence, honey bee population decline has been linked to global food security [3]. As the global population continues to increase, our dependence on pollination services is also expected to grow [3].

Research into the factors that influence honey bee health has focused extensively on pesticides; however, there may be impacts of metals such as aluminum (Al) on foraging and mortality. Al can become bioavailable in soil as a result of acidification or poor mining practices [4,5,6]. In North America, a wide range of aluminum levels have been reported in both produce and pollen ranging from 0.05 to 670 mg/L [7]. Bumble bees have been determined to consume aluminum through the pollen they forage on, which leads to exposure of the whole hive through the contaminated pollen they bring back to the hive [8]. Later, the aluminum can be detected in the stored honey that the bumble bees use in the hive, which can lead to chronic exposure to aluminum [8].

Aluminum toxicity impacts the cholinergic system by altering the enzyme activity of acetylcholinesterase (AChE) in the brain [9,10,11,12]. Disruption of this system is associated with impairments in motor functioning, learning, and memory [13,14,15]. Inhibition of AChE can also result in physiological effects such as spasms, hyperactivity, and erratic movement [15,16]. Hyperactivity can disrupt the diurnal circadian rhythms of forager bees, which may cause them to miss key foraging times for specific plants [17].

The ingestion of aqueous aluminum has been determined to affect floral foraging decisions made by honey bees [17,18]. Bees that were fed an aluminum solution exhibited reduced flexibility to changing floral environments [17,18]. In these studies, the bees showed a reduced likelihood of selecting a high caloric value reward following a single exposure to a relatively low concentration of aluminum which may be detrimental to honey bee survival. Since eusocial forager honey bees supply food for the winter stores, choosing poorer resources may impact hive health. Honey bees have also been shown to have altered AChE and dose-dependent behavioral outcomes following exposure to aluminum trichloride [9].

In a study conducted in Turkey, researchers detected aluminum levels in honey samples ranging from 0.775 μg/g to 155.586 μg/g [19]. A study in Brazil reported mean aluminum pollen content levels of 96.6 μg/g [20]. Researchers finding aluminum in honey suggest that bees are consuming pollen or nectar that is contaminated with this metal and bring it back to the hive where it is made into honey, stored, and fed to larvae. In bumble bees, larval accumulations from adult food transfer have been recorded at 13.4–193.4 μg/g dry weight [8]. Honey bees use their honey stores throughout the winter, which means the bees would be consuming the contaminated food for an extended period.

Researchers have discovered evidence that silicon and silicates may play a protective role against the detrimental impact of aluminum by binding the metal with oxygen or silicate (SiO_4_) thereby preventing absorption of the metal [21,22,23,24]. Oligomeric silica has a high affinity for aluminum and was determined to reduce the amount of aluminum in the gastrointestinal tract following oral consumption [24]. Oral silicon supplementation was determined to also limit oral aluminum absorption and retention in rats, mice, and humans [22,25,26]. Preventing the deleterious effects of aluminum on honey bee movement and circadian rhythmicity is crucial to ensuring hive health and survival, since both can impact foraging and winter food storage [17].

The main purpose of this study is to explore whether dissolved silica in Fiji water can be used as a preventative measure against aluminum chloride (AlCl3) toxicity in honey bees. Fiji water was chosen because it is a water-soluble and bioavailable source of silica. If the silica in Fiji water is determined to have protective effects, then bee keepers can use this water in hive feeders to supplement bees in areas with high levels of aluminum.

Bees were exposed to varying levels of an aqueous AlCl3 solution, and their mortality and activity rates were recorded. Depending on the treatment group, the bee was either exposed to an AlCl3 solution before or after being exposed to Fiji water, AlCl3 before or after deionized water (DI), DI before or after Fiji water, Fiji water only, or DI only. We expect that the bees exposed to Fiji water before AlCl3 will receive a protective effect against effects of aluminum trichloride from silica uptake. These bees will exhibit lower mortality rates compared to bees that were exposed to AlCl3 before silica and bees that were exposed to AlCl3 before or after DI water. Additionally, we hypothesize that the bees that receive the Fiji water first will exhibit activity and rhythmicity levels closer to those of the controls than the bees that receive the AlCl3 first or the bees that receive DI water before or after AlCl3.

## 2. Materials and Methods

### 2.1. Study Subjects

A total of 850 honey bees from three hives were used for this study. Only forager bees were selected, as they are assumed to be around the same age (20 + days old: [27,28]). Bees were captured in 15 mL falcon tubes off a feeder that contained 1 M sucrose solution (see Delkash-Roudsari et al. [29]). Each tube lid contained a patty of an approximately 40:60 mixture of honey and sucrose (bee candy) [17]. This mixture was covered with a piece of cheese cloth so that the bees would not become adhered to the food source. Following collection, bees were transferred back to the laboratory where the tubes were placed in the monitor apparatus (Trikinetics Inc. Waltham, MA, USA).

### 2.2. Monitors System

The monitor apparatus (TriKinetics Inc. Waltham, MA, USA) records the activity data for up to 32 honey bees in individual 15 mL falcon tubes (Globe Scientific, Mahwah, NJ, USA). The data collected by this apparatus allows researchers to conduct chronic exposure studies on the effects of toxicants on activity, circadian rhythmicity, and mortality [17,29]. Each falcon tube has aeration holes, one of which was fitted with a 0.25 cm × 3 cm piece of filter paper so that the bees could consume the solution from the filter paper. The other end of the filter paper was extended down into a hole in a 40 cm long × 1 cm diameter piece of Chlorinated Polyvinyl Chloride (CPVC) pipe (Silver-Line Plastics, Lawton, OK, USA).that was attached to the monitor apparatus, so that it was submerged in the solution (see Figures S1 and S2; for additional images of this system, see [17]). For the monitors that were used for the treatment conditions, one end of each CPVC pipe had a 1.9 cm PVC elbow to allow for easier flushing when changing between aluminum solution and the Fiji water. Each CPVC pipe was filled with 40 mL of fluid based on the treatment group and then the monitor was placed in an incubator (24 h darkness, 35 ± 2 °C, and 40% humidity). The CPVC pipes each provided water to 8 bees through filter paper strips, which enabled the bees to receive their respective treatment (Fiji water, aqueous AlCl3, or deionized water).

In the monitor system, each falcon tube was surrounded by six photocells (Trikientics Inc., Waltham, MA, USA) which recorded when the bee crossed the centerline. The monitor records the bee as deceased if she does not cross the centerline for 24 h. Throughout the duration of this study, bees were kept in darkness, except for the replacement of food-filled tube lids or topping off/changing of solutions during which the bees were exposed to red light [17]. The exposure to red light is not expected to have influenced their behavior as bees cannot see in the red spectrum [30]. Every 48 h ± 8 h the CPVC pipes were topped off with up to 20 mL of the designated treatment solution.

### 2.3. Chronic Exposure Dosing

The monitor system automatically collects data per bee per minute, which is ideal for studying chronic exposure to pesticides and metal toxicity [17,29,31]. The apparatus that we used can be viewed at the following link on the Trikinetics website. Bees in the experimental treatment groups were exposed to a solution of deionized water (DI) mixed with AlCl3 (25 mg/L, 75 mg/L, 150 mg/L, or 300 mg/L) (Table 1). The control bees were exposed to DI water only. Additional experimental control bees received Fiji water only. The Fiji water that we used contained 93 mg/L of silica, 18 mg/L calcium, 15 mg/L magnesium, and had a pH of 7.7 according to the following website: https://www.fijiwater.com (15 September 2022) There were also bees that were exposed to DI water before (i.e., Experimental Design Control bees) or after Fiji water (i.e., Counterbalanced Experimental Design Control bees).

Five times the concentration of AlCl3 (Sigma-Aldrich, St. Louis, MO, USA, 99%) (listed above) was added to account for the mass of the chloride which is expected to be inert [17]. The concentrations used were based on those used in previous studies, which had investigated a range of aluminum that had been detected in pollen [17]. In our experiment, water was our primary exposure route, which does not allow us to make direct comparisons with pollen aluminum concentration levels. However, they can be used as an indicator due to the similarities between pollen and water concentrations (see Chicas-Mosier et al. [17]). Bees were either exposed to Fiji water first for four days, DI water first for four days, or an AlCl3 solution first and then Fiji water, DI water first and then an AlCl3 solution, DI water only, or Fiji water only (Table 1). Every four days, the bees had their solutions changed, except for the DI and Fiji water only controls. Additionally, one group of bees was exposed to Fiji water and then DI water, while another was exposed to DI water and then Fiji water following the same four-day switching process. Each treatment group was paired with a simultaneous (±2 days) DI control monitor (4 experimental setups between 1 June and 30 July 2020, and 6 experimental setups between 28 February and 17 September 2022). Data collection occurred until all bees were deceased or for a maximum of 16 days.

### 2.4. Data Analysis

Each experiment was conducted over a 16-day period with a DI control group run simultaneously to account for periodic random effects not related to the actual treatment effect that was tested. The experiment was replicated twice in two different time periods: over the years of 2020 (4 experimental setups between 1 June and 30 July) and 2022 (6 experimental setups between 28 February and 17 September). As a result, we analyzed the data for differences in the control treatments as well as the experimental treatments between the two years and each experiment cycle.

Data analyses were conducted using SPSS 24 IBM (Armonk, NY, USA). One-way analysis of covariance (ACOVA) tests were run to determine whether there were significant differences between the nine control groups for year with experiment cycle as a covariate to test circadian rhythmicity, average daily activity, and mortality while controlling for the year. There were significant variations between the nine control monitors for average daily activity with the bees in the control treatment tested in 2020 (*M* = 1266.21, *SD* = 1638.79) having significantly higher average daily activity rates compared to the control treatment bees tested in 2022 (*M* = 657.65, *SD* = 1384.42), (*F*(1, 5250) = 50.29, *p* < 0.001). There were significant variations between the nine control monitors for rhythmicity with the control bees tested in 2020 (*M* = −8.96, *SD* = 61.54) having significantly higher average daily activity rates compared to the control bees tested in 2022 (*M* = −321.12, *SD* = 585.02), (*F*(1, 15,176) = 1457.65, *p* < 0.001). Additionally, there was a main effect for the experiment cycle covariate on circadian rhythmicity (*F*(1, 15,176) = 301.76, *p* < 0.001). There were no significant differences in mortality between the control bees tested in 2020 (*M* = 8.51, *SD* = 3.72) and the control bees tested in 2022 (*M* = 9.52, *SD* = 3.89) when controlling for experiment cycle (*F*(2, 215) = 2.62, *p* = 0.075). To account for this variation, standardization of the experimental groups was conducted by subtracting the appropriate hourly control from the experimental value, thereby creating a baseline value of zero for all comparisons [29].

Rhythmicity was operationally defined as deviation from the zero-baseline across a day: night shift (0600–1800 h:1800–0600 h). Activity was defined as average deviation from zero-baseline across the total number of days alive per honey bee. One-way ANCOVA tests were run with year as a covariate and experiment cycle as a nested covariate to compare the rhythmicity, average daily activity, and average number of days alive of the experimental groups with their respective DI–aluminum or aluminum–DI groups. Additionally, honey bee survival data were compared to controls using Mantel–Cox log-rank mortality tests. One-way ANCOVA tests with year as a covariate and experiment cycle as a nested covariate were also conducted to compare all groups for rhythmicity, average daily activity, and average number of days alive. Additional one-sample *t*-tests were conducted to compare average daily activity and rhythmicity rates of the experimental groups to a zero-baseline (representing the controls).

## 3. Results

### 3.1. Rhythmicity Data

The one-way ANCOVA tests that compared all of the groups’ average 24 h rhythmicity with year as a covariate and experiment cycle as a nested covariate was significant, *F*(22, 19,839) = 590.52, *p* < 0.001. Overall, the analysis showed that there were significant differences in average 24 h rhythmicity between the groups. Post hoc tests revealed significantly higher rhythmicity rates in AlCl3 before and after Fiji groups compared to the AlCl3 before and after DI groups. There were significant main effects for experimental group [*F*(20, 19,839) = 642.33, *p* < 0.001], year [*F*(1, 19,839) = 1531.60, *p* < 0.001], and experiment cycle, *F*(1, 19,839) = 167.37, *p* < 0.001. Post hoc comparisons were run using a Bonferroni correction to compare the different experimental groups.

**Figure 1:**
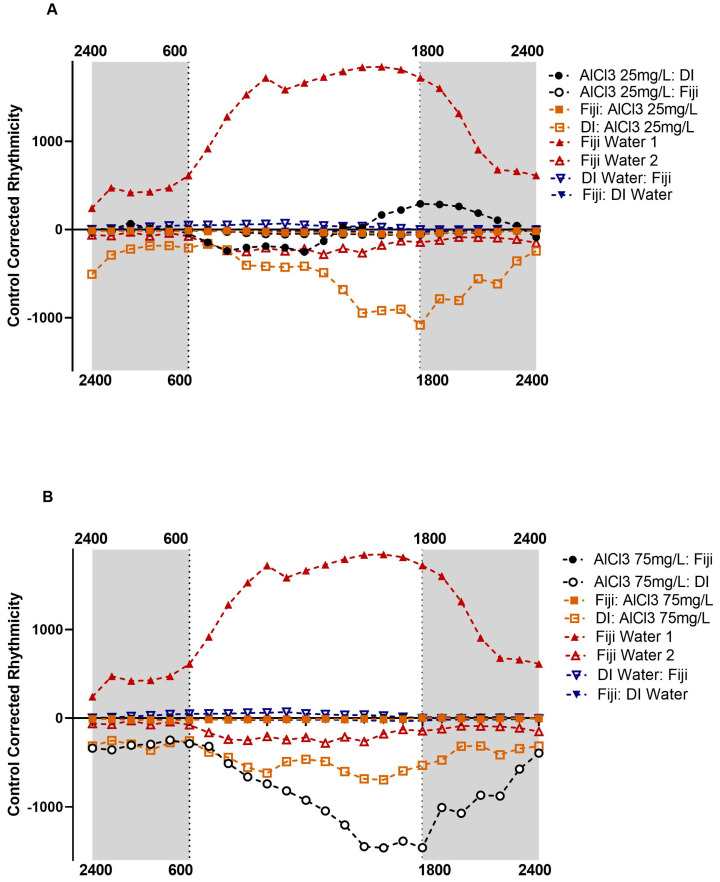

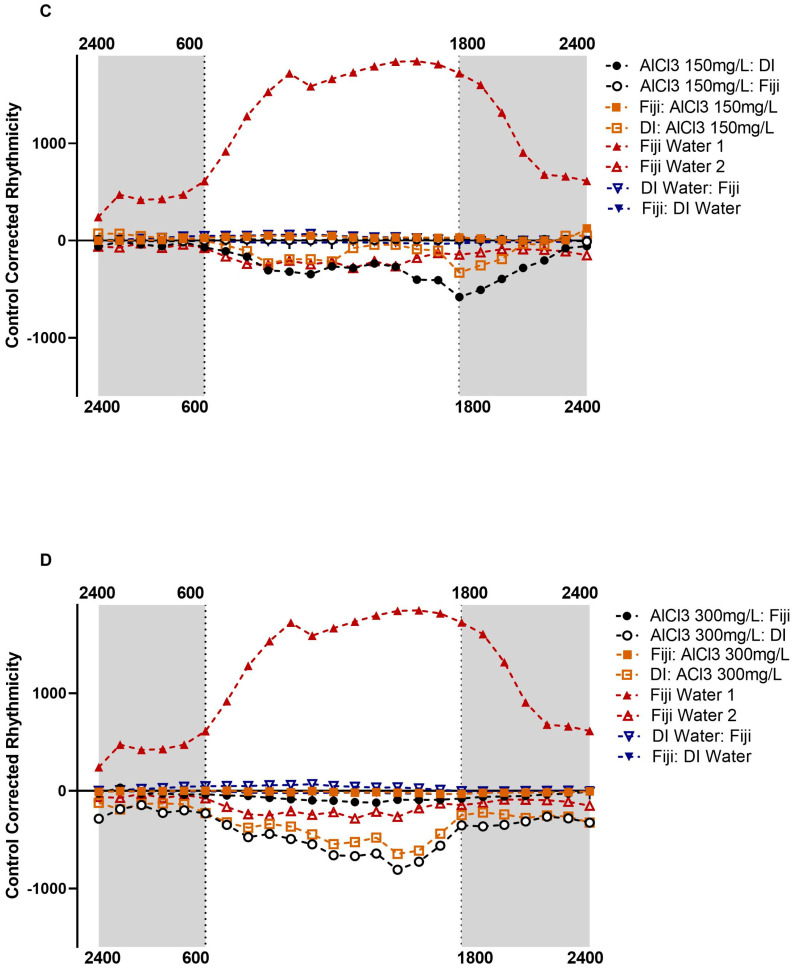
The average 24 h activity by exposure group for bees by aluminum concentration. The zero line represents the baseline control data. Although the bees were kept in 24 h darkness, the shaded sections mark nighttime hours (1800–0600 h). (**A**) Activity for AlCl3 25 mg/L and respective control group bees. (**B**) Activity for AlCl3 75 mg/L and respective control bees. (**C**) Activity for AlCl3 150 mg/L and respective control bees. (**D**) Activity for AlCl3 300 mg/L and respective control bees.

The post hoc comparisons of the average 24 h rhythmicity for the AlCl3 at 25 mg/L concentration groups showed significant differences in rhythmicity for the AlCl3 at 25 mg/L before (AlCl3 25 mg/L→Fiji) and after Fiji (Fiji→AlCl3 25 mg/L) and the AlCl3 at 25 mg/L before (AlCl3 25 mg/L→DI) and after DI water (DI→AlCl3 25 mg/L) groups, as shown in Tables S1 and S2 and Figure 1A. The AlCl3 25 mg/L→Fiji group (*M* = −30.61, *SD* = 48.51) had significantly higher rhythmicity levels compared to the AlCl3 25 mg/L→DI (*M* = 7.61, *SD* = 809.75, *p* < 0.001) and DI→AlCl3 25 mg/L (*M* = −490.09, *SD* = 579.84, *p* < 0.001) groups, as shown in Table S1. The AlCl3 25 mg/L→DI group had significantly lower rhythmicity levels compared to the Fiji→AlCl3 25 mg/L group (*M* = −27.51, *SD* = 70.41, *p* < 0.001) and significantly higher rhythmicity levels compared to the DI→AlCl3 25 mg/L group (*p* < 0.001), as shown in Tables S1 and S2, Figure 1A. The Fiji→AlCl3 25 mg/L group had significantly higher rhythmicity rates compared to the DI→AlCl3 25 mg/L group (*p* < 0.001). The only comparison that was not significantly different was between the AlCl3 25 mg/L→Fiji group and the Fiji→AlCl3 25 mg/L group, *p* = 1.000. Overall, there was evidence of an effect of aluminum on rhythmicity with significant differences between the groups that received DI water and Fiji water. The bees that received Fiji before or after AlCl3 25 mg/L exhibited significantly higher rates of rhythmicity compared to the bees that received aluminum before or after DI water. There was not a significant difference in rhythmicity between the bees that received AlCl3 25 mg/L before Fiji and the bees that received aluminum after Fiji.

The post hoc comparisons of the average 24 h rhythmicity for the AlCl3 at 75 mg/L groups showed significant differences in rhythmicity for the AlCl3 at 75 mg/L before (AlCl3 75 mg/L→Fiji) and after Fiji (Fiji→AlCl3 75 mg/L) and the AlCl3 at 75 mg/L before (AlCl3 75 mg/L→DI) and after DI water groups (DI→AlCl3 75 mg/L), as shown in Tables S1 and S2 and Figure 1B. The AlCl3 75 mg/L→Fiji group (*M* = 0.59, *SD* = 45.93) had significantly higher rhythmicity levels compared to the AlCl3 75 mg/L→DI (*M* = −778.20, *SD* = 495.16, *p* < 0.001) and DI→AlCl3 75 mg/L (*M* = −436.52, *SD* = 618.91) groups (*p* < 0.001), as shown in Table S1. The AlCl3 75 mg/L→DI group had significantly lower rhythmicity levels compared to the Fiji→AlCl3 75 mg/L (*M* = −11.56, *SD* = 36.66, *p* < 0.001) and the DI→AlCl3 75 mg/L groups (*p* < 0.001), as shown in Tables S1 and S2 and Figure 1B. The Fiji→AlCl3 75 mg/L group had significantly higher rhythmicity rates compared to the DI→AlCl3 75 mg/L group, *p* < 0.001. There was no significant difference between the AlCl3 75 mg/L→Fiji and Fiji→AlCl3 75 mg/L groups, *p* = 1.000. Overall, there was evidence of an effect of aluminum on rhythmicity with significant differences between the groups that received DI water and Fiji water at the AlCl3 75 mg/L concentration. The bees that received Fiji before or after AlCl3 75 mg/L exhibited significantly higher rates of rhythmicity compared to the bees that received aluminum before or after DI water. There was not a significant difference in rhythmicity between the bees that received AlCl3 75 mg/L before Fiji and the bees that received aluminum after Fiji.

The post hoc comparisons of the average 24 h rhythmicity for the AlCl3 150 mg/L groups showed significant differences in rhythmicity for the AlCl3 at 150 mg/L before (AlCl3 150 mg/L→Fiji) and after (Fiji→AlCl3 150 mg/L) Fiji and the AlCl3 at 150 mg/L before (AlCl3 150 mg/L→DI) and after (DI→AlCl3 150 mg/L) DI water groups, as shown in Tables S1 and S2 and Figure 1C. The AlCl3 150 mg/L→Fiji group (*M* = 7.19, *SD* = 54.58) had significantly higher rhythmicity levels compared to the AlCl3 150 mg/L→DI (*M* = −227.77, *SD* = 315.57, *p* < 0.001) and DI→AlCl3 150 mg/L (*M* = −77.56, *SD* = 401.39) groups (*p* < 0.001), as shown in Table S1. The AlCl3 150 mg/L→Fiji group had significantly lower rhythmicity rates compared to the Fiji→AlCl3 150 mg/L (*M* = 10.97, *SD* = 71.49), *p* < 0.001. The AlCl3 150 mg/L→DI group had significantly lower rhythmicity levels compared to the Fiji→AlCl3 150 mg/L (*p* < 0.001) and DI→AlCl3 150 mg/L groups (*p* < 0.001), as shown in Tables S1 and S2 and Figure 1C. The AlCl3 150 mg/L→Fiji group had significantly higher rhythmicity rates compared to the DI→AlCl3 150 mg/L group, *p* < 0.001. Overall, there was evidence of an effect of aluminum on rhythmicity with significant differences between the groups that received DI water and Fiji water at the AlCl3 150 mg/L concentration. The bees that received Fiji before or after AlCl3 150 mg/L exhibited significantly higher rates of rhythmicity compared to the bees that received aluminum before or after DI water. There was a significant difference in rhythmicity between the bees that received AlCl3 150 mg/L before Fiji and the bees that received aluminum after Fiji. Bees that received AlCl3 150 mg/L before Fiji water had significantly lower rhythmicity rates compared to bees that received aluminum after Fiji water.

The post hoc comparisons of the average 24 h rhythmicity for the AlCl3 300 mg/L groups showed significant differences in rhythmicity for the AlCl3 at 300 mg/L before (AlCl3 300 mg/L→Fiji) and after Fiji (Fiji→AlCl3 300 mg/L) and the AlCl3 at 300 mg/L before (AlCl3 300 mg/L→DI) and after DI water (DI→AlCl3 300 mg/L) groups. The AlCl3 300 mg/L→Fiji group (*M* = −61.33, *SD* = 40.28) had significantly higher rhythmicity levels compared to the AlCl3 300 mg/L→DI (*M* = −411.91, *SD* = 408.33, *p* < 0.001) and DI→AlCl3 300 mg/L (*M* = −327.60, *SD* = 464.39) groups (*p* < 0.001), as shown in Table S1 and Figure 1D. The AlCl3 300 mg/L→Fiji group had significantly lower rhythmicity rates compared to the Fiji→AlCl3 300 mg/L (*M* = −11.62, *SD* = 41.48) group, *p* < 0.001. The AlCl3 300 mg/L→DI group had significantly lower rhythmicity levels compared to the Fiji→AlCl3 300 mg/L (*p* < 0.001) and the DI→AlCl3 300 mg/L groups (*p* = 0.02), as shown in Tables S1 and S2 and Figure 1A. The Fiji→AlCl3 300 mg/L group had significantly higher rhythmicity rates compared to the DI→AlCl3 300 mg/L group (*p* < 0.001). Overall, there was evidence of an effect of aluminum on rhythmicity with significant differences between the groups that received DI water and Fiji water at the AlCl3 300 mg/L concentration. The bees that received Fiji before or after AlCl3 300 mg/L exhibited significantly higher rates of rhythmicity compared to the bees that received aluminum before or after DI water. There was a significant difference in rhythmicity between the bees that received AlCl3 300 mg/L before Fiji and the bees that received aluminum after Fiji. Bees that received AlCl3 300 mg/L before Fiji water had significantly lower rhythmicity rates compared to bees that received aluminum after Fiji water.

Two additional one-way ACOVA tests were conducted, one for daytime activity and one for nighttime activity. The one-way ANCOVA for daytime (0600–1800 h) rhythmicity while controlling for year and experiment cycle was significant, *F*(22, 9074) = 400.30, *p* < 0.001). There were significant main effects for year [*F*(1, 9074) = 1082.39, *p* < 0.001], experiment cycle [*F*(1, 9074) = 112.56, *p* < 0.001], and aluminum concentration, *F*(20, 9074) = 436.86, *p* < 0.001. Overall, year, experiment cycle, and aluminum concentration significantly impacted daytime rhythmicity rates. The Fiji 1, DI water before and after Fiji water, Fiji→AlCl3 1500 mg/L, and Fiji→AlCl3 300 mg/L daytime groups exhibited no significant differences compared to the controls, as shown in Table S2 for descriptive statistics and Figure 1A–D. However, the remainder of the AlCl3 before and after Fiji and AlCl3 before and after DI water groups along with the Fiji 2 group showed significant differences compared to the DI water controls (see Table S2 for descriptive statistics and Figure 1A–D). Cumulatively, the data showed significant daytime differences between the treatment groups and the DI water control for all but five of the groups.

The one-way ANCOVA for nighttime (1800–0600 h) rhythmicity while controlling for year and experiment cycle was significant, *F*(22, 10,742) = 293.10, *p* < 0.001). There were significant main effects for year [*F*(1, 10,742) = 685.22, *p* < 0.001], experiment cycle [*F*(1, 10,742) = 82.96, *p* < 0.001], and aluminum concentration, *F*(20, 10,742) = 314.98, *p* < 0.001. Overall, year, experiment cycle, and aluminum concentration significantly impacted nighttime rhythmicity rates. The Fiji 1, DI water before and after Fiji water, Fiji→AlCl3 150 mg/L, and Fiji→AlCl3 300 mg/L nighttime groups exhibited no significant differences compared to the controls, as shown in Table S2 for descriptive statistics and Figure 1A–D. However, the remainder of the AlCl3 before and after Fiji and AlCl3 before and after DI water groups along with the Fiji 2 group showed significant differences to controls (see Table S2 for descriptive statistics and Figure 1A–D). Cumulatively, the data showed significant nighttime differences between the treatment groups and the DI water control for all but five of the groups.

Additional single sample *t*-test analyses were conducted with the daytime and nighttime data to compare each of the experimental groups to a zero-baseline. Overall, 14 out of the 21 groups had significantly lower rhythmicity levels during the daytime compared to the zero-baseline, Tables S1 and S2. When AlCl3 at 75 mg/L was introduced prior to Fiji water (AlCl3 75 mg/L→Fiji) and AlCl3 at 25 mg/L was introduced prior to DI water (AlCl3 25 mg/L→DI) groups exhibited no significant differences in daytime rhythmicity levels from the zero-baseline. During the daytime, Fiji water 1, DI Water before Fiji water, and AlCl3 at 150 mg/L before and after Fiji groups all had significantly higher rhythmicity rates compared to the zero-baseline.

During the nighttime, 13 out of the 21 groups had significantly lower rhythmicity levels compared to the zero-baseline, as shown in Table S1. The same four groups, Fiji water 1, DI Water before Fiji, AlCl3 at 150 mg/L before and after Fiji water, had significantly higher nighttime rhythmicity rates compared to the zero-baseline. Additionally, the same two groups, AlCl3 at 75 mg/L before Fiji water and AlCl3 at 25 mg/L before DI water, exhibited no significant differences in daytime rhythmicity levels from the zero-baseline. A third group, AlCl3 at 150 mg/L after DI water, exhibited no significant differences in nighttime rhythmicity compared to the zero-baseline.

In comparison, 12 out of the 20 groups had significantly lower rhythmicity levels for their average 24 h activity levels. The same four groups from the daytime and nighttime, Fiji water 1, DI water before Fiji, and AlCl3 at 150 mg/L before and after Fiji water groups had significantly higher rhythmicity levels compared to the zero-baseline. The DI water after Fiji water, and AlCl3 at 300 mg/L after Fiji water groups all had significantly higher rhythmicity rates compared to the zero-baseline. The same two groups, AlCl3 at 75 mg/L before Fiji water and AlCl3 at 25 mg/L before DI water, exhibited no significant differences in rhythmicity levels during their average 24 h of activity compared to the zero-baseline.

### 3.2. Average Daily Activity

**Figure 2:**
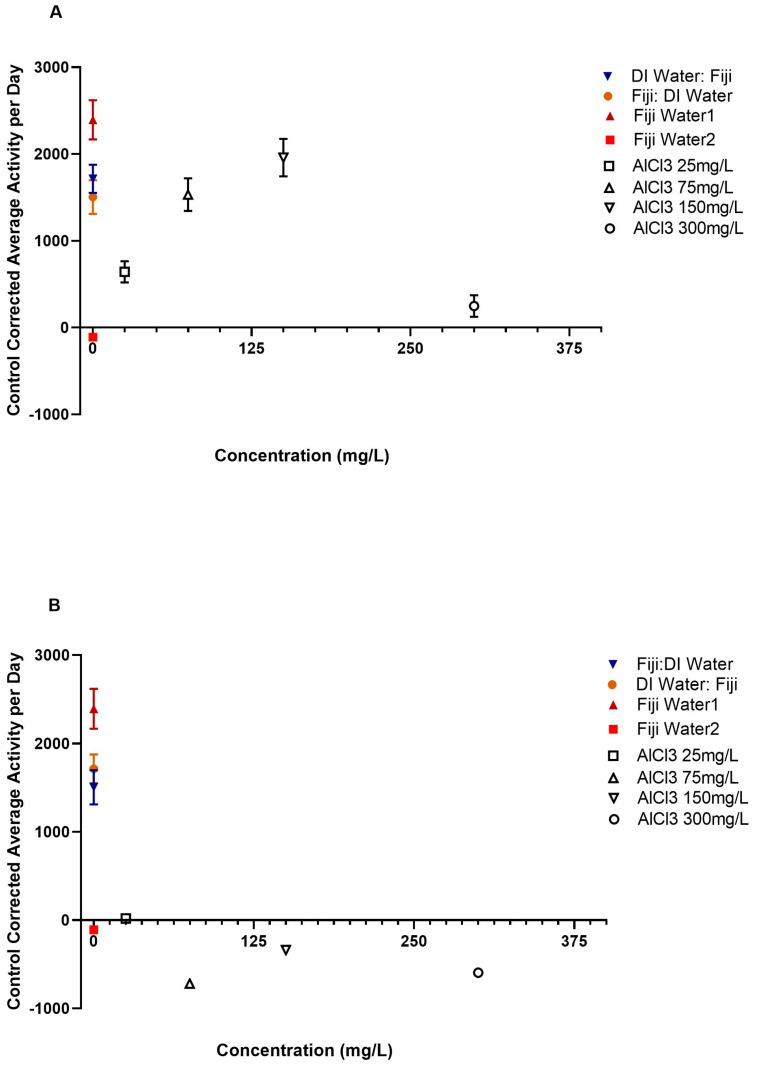

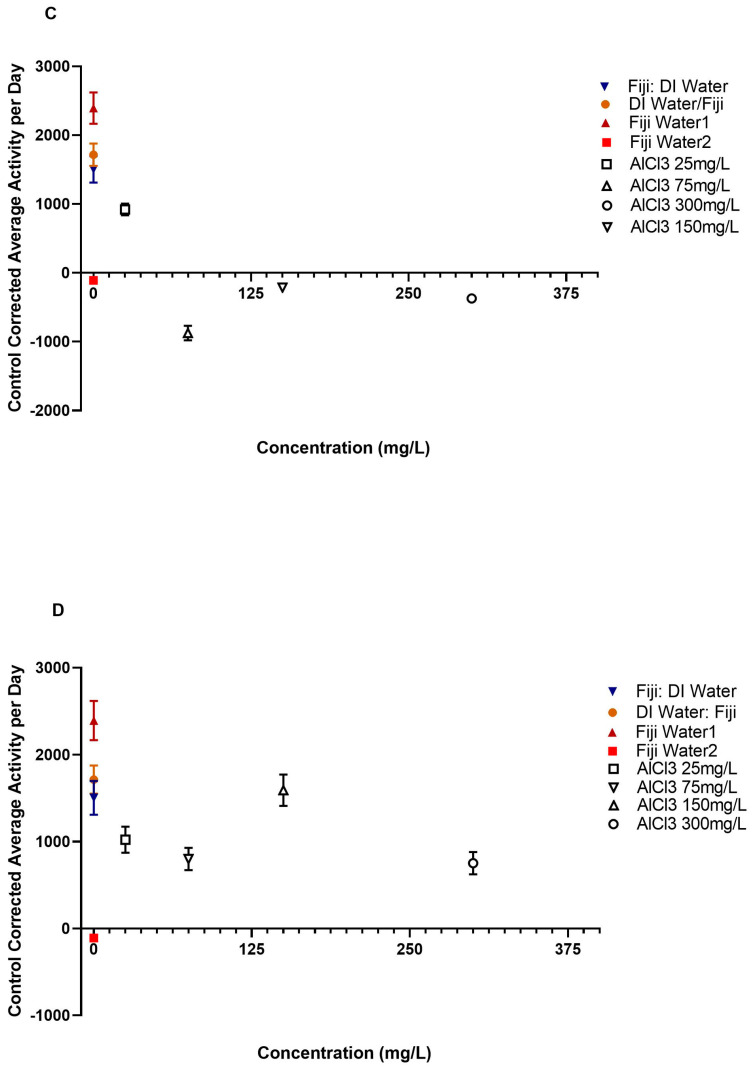
The overall daily activity for honey bees by first exposure group with standard error of the mean bars. The zero line represents the baseline control data. (**A**) Activity for AlCl3→Fiji groups compared to Fiji water, DI water→Fiji, and Fiji→DI water control group bees. (**B**) Activity for AlCl3→ DI water groups compared to respective Fiji water, DI water→Fiji, and Fiji→DI water control group bees. (**C**) Activity for DI water→ AlCl3 groups compared to respective Fiji water, DI water→Fiji, and Fiji→DI water control group bees. (**D**) Activity for Fiji→ AlCl3 compared to respective Fiji water, DI water→Fiji, and Fiji→DI water control group bees.

The one-way ANCOVA test that compared all of the groups’ average daily activity with year as a covariate and experiment cycle as a nested covariate was significant, *F*(22, 14,435) = 131.58, *p* < 0.001. Overall, the analysis showed that there were significant differences in average daily activity between the groups. Post hoc tests revealed significantly higher average daily activity rates between a portion of the AlCl3 before and after Fiji groups compared to the AlCl3 before and after DI groups. There were significant main effects for experimental group [*F*(20, 14,435) = 66.39, *p* < 0.001] and year, *F*(1, 14,435) = 33.02, *p* < 0.001. There was no significant main effect for the experiment cycle, *F*(1, 14,435) = 0.09, *p* = 0.759. Post hoc comparisons were run using a Bonferroni correction to compare the different groups, as shown in Tables S3 and S4 and Figure 2A–D.

Post hoc comparisons for the AlCl3 25 mg/L groups showed significant differences in average daily activity between the AlCl3 before DI and both the AlCl3 after Fiji and after DI groups after controlling for year and experiment cycle, as shown in Tables S3 and S4 and Figure 2A–D. The AlCl3 25 mg/L→DI (*M* = −18.34, *SD* = 1116.22) had significantly higher average daily activity rates compared to the DI→AlCl3 25 mg/L (*M* = −443.90, *SD* = 921.70, *p* = 0.005) group. The Fiji→AlCl3 25 mg/L group (*M* = −1022.76, *SD* = 2546.14) had significantly higher average daily activity levels compared to the DI→AlCl3 25 mg/L group, *p* < 0.001. There were no significant differences between the AlCl3 25 mg/L→Fiji group and the AlCl3 at 25 mg/L before (*p* = 1.00) or after DI (*p* = 0.12) groups, as seen in Tables S3 and S4 and Figure 2A–D. Additionally, there was no significant difference between the AlCl3 before and after Fiji groups, *p* = 1.00. There also was no significant difference between the AlCl3 25 mg/L→DI group and the Fiji→AlCl3 25 mg/L group, *p* = 0.922. Overall, there was only a significant difference between the AlCl3 before and after DI groups and the AlCl3 after Fiji groups with the aluminum after DI group. The AlCl3 25 mg/L before Fiji group had significantly higher average daily activity rates compared to the AlCl3 after DI group. Additionally, the AlCl3 25 mg/L after Fiji group had significantly higher average daily activity levels compared to the AlCl3 25 mg/L after DI group. There were no significant differences between any of the other AlCl3 25 mg/L before and after Fiji groups or the other AlCl3 25 mg/L before and after DI water and AlCl3 25 mg/L before and after Fiji water groups.

There were significant differences between the AlCl3 at 75 mg/L before and after DI and the AlCl3 75 mg/L before and after Fiji groups in average daily activity, as shown in Tables S3 and S4 and Figure 2A–D. The AlCl3 75 mg/L→Fiji group (*M* = 1532.74, *SD* = 2707.88) had significantly higher average daily activity rates compared to the AlCl3 at 75 mg/L before (*M* = −718.89, *SD* = 740.99, *p* < 0.001) and after DI (*M* = −878.82, *SD* = 3402.04, *p* < 0.001) groups, and Fiji→AlCl3 75 mg/L (*M* = 801.10, *SD* = 2051.49, *p* < 0.001) groups. The AlCl3 300 mg/L→DI group had significantly lower average daily activity levels compared to the Fiji→AlCl3 75 mg/L group, *p* < 0.001. There was not a significant difference between the AlCl3 at 75 mg/L before and after DI groups (*p* = 1.00), as seen in Tables S3 and S4 and Figure 2A–D. The AlCl3 at Fiji→AlCl3 75 mg/L group had a significantly higher average daily activity rate compared to the DI→AlCl3 300 mg/L group, *p* < 0.001. Overall, there were significant differences between the AlCl3 before and after DI groups and the AlCl3 before and after Fiji groups. There was also a significant difference between the AlCl3 before and after Fiji water groups. The AlCl3 75 mg/L before Fiji group had significantly higher average daily activity rates compared to the AlCl3 75 mg/L before and after DI groups. Additionally, the AlCl3 75 mg/L after Fiji group had significantly higher average daily activity levels compared to the AlCl3 75 mg/L before and after DI groups. The AlCl3 75 mg/L before Fiji group had significantly higher average daily activity rates compared to the AlCl3 75 mg/L after Fiji group. There were no significant differences between the AlCl3 75 mg/L before and after DI water groups.

There were also significant differences between the AlCl3 at 150 mg/L before and after DI and the AlCl3 at 150 mg/L before and after Fiji groups in average daily activity, as shown in Tables S3 and S4 and Figure 2A–D. The AlCl3 at 150 mg/L before Fiji group (*M* = 1958.36, *SD* = 3068.83) had significantly higher average daily activity rates compared to the AlCl3 at 150 mg/L before DI (*M* = −338.41, *SD* = 813.07, *p* < 0.001) and AlCl3 at 150 mg/L after DI (*M* = −214.79, *SD* = −7.83) groups, *p* < 0.001. There was not a significant difference between the AlCl3 at 150 mg/L before Fiji group and the AlCl3 at 150 mg/L after Fiji group (*M* = 1592.61, *SD* = 3151.64), *p* = 1.00. The AlCl3 at 150 mg/L before DI group had significantly lower average daily activity levels compared to the AlCl3 at 150 mg/L after Fiji group, *p* < 0.001. There was not a significant difference between the AlCl3 at 150 mg/L before DI group and the AlCl3 at 150 mg/L after DI group (*p* = 1.00), as shown in Tables S3 and S4 and Figure 2A–D. The AlCl3 at 150 mg/L after Fiji group has a significantly higher average daily activity rate compared to the AlCl3 at 150 mg/L after DI group, *p* < 0.001. Overall, there were significant differences between the AlCl3 before and after DI groups and the AlCl3 before and after Fiji groups. There was also a significant difference between the AlCl3 before and after Fiji water groups. The AlCl3 150 mg/L before Fiji group had significantly higher average daily activity rates compared to the AlCl3 150 mg/L before and after DI groups. Additionally, the AlCl3 150 mg/L after Fiji group had significantly higher average daily activity levels compared to the AlCl3 150 mg/L before and after DI group. There were no significant differences between the AlCl3 150 mg/L before and after DI water groups and the AlCl3 150 mg/L before and after Fiji groups.

The majority of the AlCl3 at 300 mg/L before and after DI and the AlCl3 at 300 mg/L before and after Fiji groups exhibited no significant differences in average daily activity rates, as shown in Tables S3 and S4. There was only a significant difference between the AlCl3 at 300 mg/L before DI group (*M* = −595.04, *SD* = 809.19) and the ALCL3 at 300 mg/L after Fiji group (*M* = 752.48, *SD* = 2037.25) in average daily activity, as shown in Tables S3 and S4 and Figure 2A–D. The AlCl3 at 300 mg/L after DI group (*M* = −371.71, *SD* = 784.12) exhibited no significant differences in average daily activity rates compared to the AlCl3 at 300 mg/L after Fiji group, *p* = 1.00. There was not a significant difference between the AlCl3 at 300 mg/L before Fiji group (*M* = 248.77, *SD* = 1550.81) and the AlCl3 at 300 mg/L before and after DI groups, *p* = 1.00. There was also no significant difference between the AlCl3 at 300 mg/L before Fiji group and the AlCl3 at 300 mg/L after Fiji group. Additionally, there was no significant difference between the AlCl3 at 300 mg/L before DI group and the AlCl3 at 300 mg/L after DI group (*p* = 1.00), as shown in Tables S3 and S4 and Figure 2A–D. There also was no significant difference between the AlCl3 at 300 mg/L after Fiji group compared to the AlCl3 at 300 mg/L after DI group, *p* = 0.08. Overall, there was only a significant difference AlCl3 300 mg/L before DI group and the AlCl3 300 mg/l after Fiji group. The AlCl3 300 mg/L before DI group had significantly lower average daily activity rates compared to the AlCl3 300 mg/L after Fiji group. There were no significant differences between any of the other AlCl3 300 mg/L before or after DI groups and the other AlCl3 300 mg/L before or after Fiji water groups. There were also no significant differences between the AlCl3 300 mg/L before and after DI water groups and the AlCl3 300 mg/L before and after Fiji groups.

Additional single sample *t*-test analyses were conducted utilizing the daytime and nighttime data to compare each of the experimental groups to a zero-baseline. The no aluminum experimental design control groups (Fiji water→DI water, DI water→Fiji water, and Fiji water 1) all had significantly higher average daily activity rates (Table S3 and Figure 2A) compared to the zero-baseline group. In comparison, the AlCl3→ DI (except for the AlCl3 25 mg/L→ DI) and DI→AlCl3 control groups along with the Fiji water 2 group had significantly lower average daily activity levels compared to the zero-baseline. Additionally, all but five of the aluminum treatments showed significantly decreased average daily activity regardless of whether the bees received Fiji water before or after exposure (Table S3 and Figure 2A–D). Bees in the AlCl3 25 mg/L→ Fiji, Fiji→ AlCl3 150 mg/L, and Fiji→ AlCl3 300 mg/L groups showed significantly higher than average daily activity rates compared to the zero-baseline (Table S3). In addition, bees in the AlCl3 75 mg/L→ Fiji group exhibited no significant difference in average daily activity compared to the zero-baseline, as shown in Table S3.

### 3.3. Mortality

**Figure 3:**
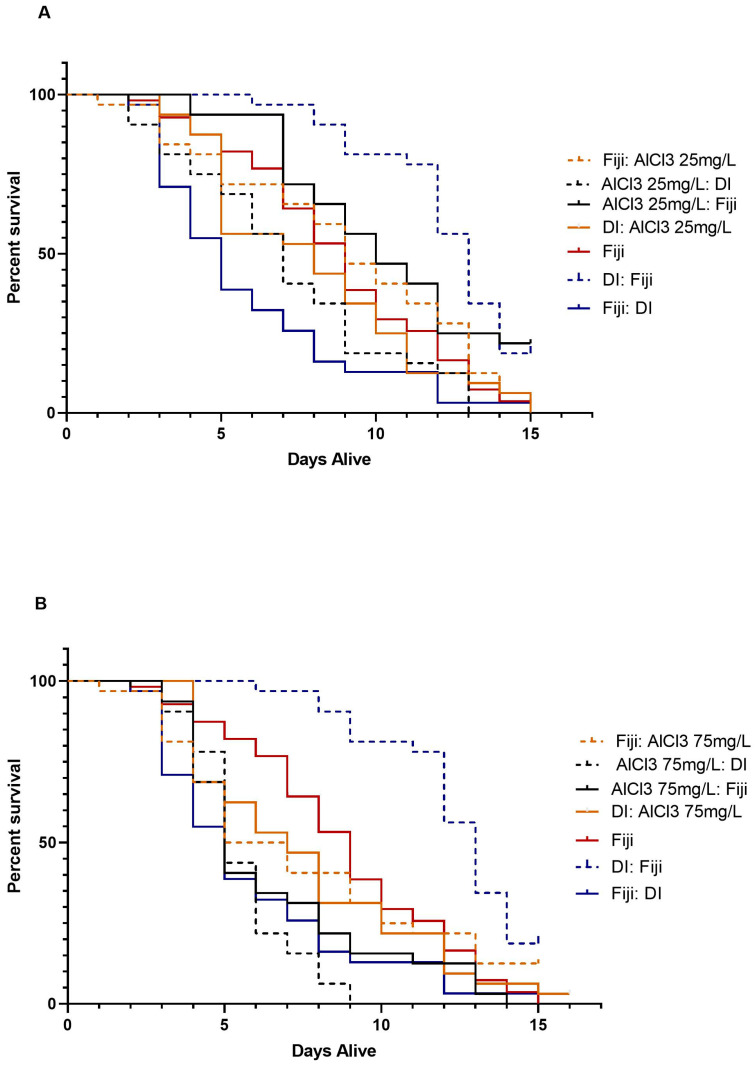

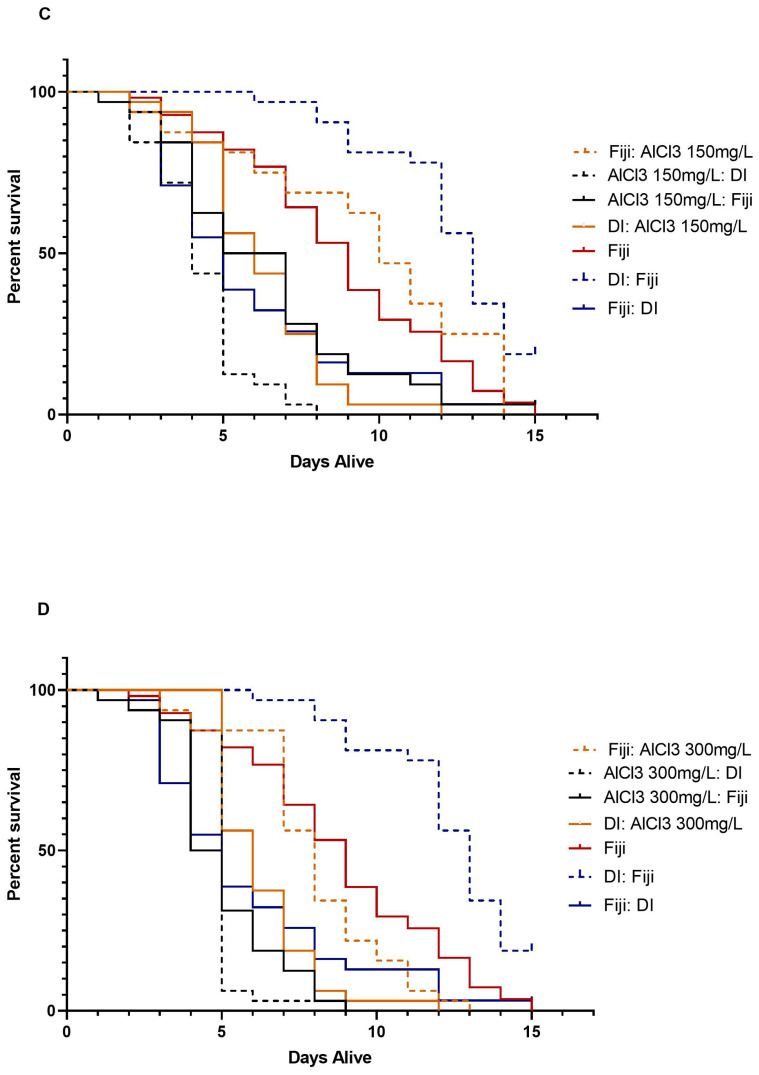
Survival curve for experimental honey bees by aluminum concentration. (**A**) Days alive for AlCl3 25 mg and respective control group bees. (**B**) Days alive for AlCl3 75 mg/L and respective control group bees. (**C**) Days alive for AlCl3 150 mg/L and respective control group bees. (**D**) Days alive for AlCl3 300 mg/L and respective control group bees.

**Figure 4:**
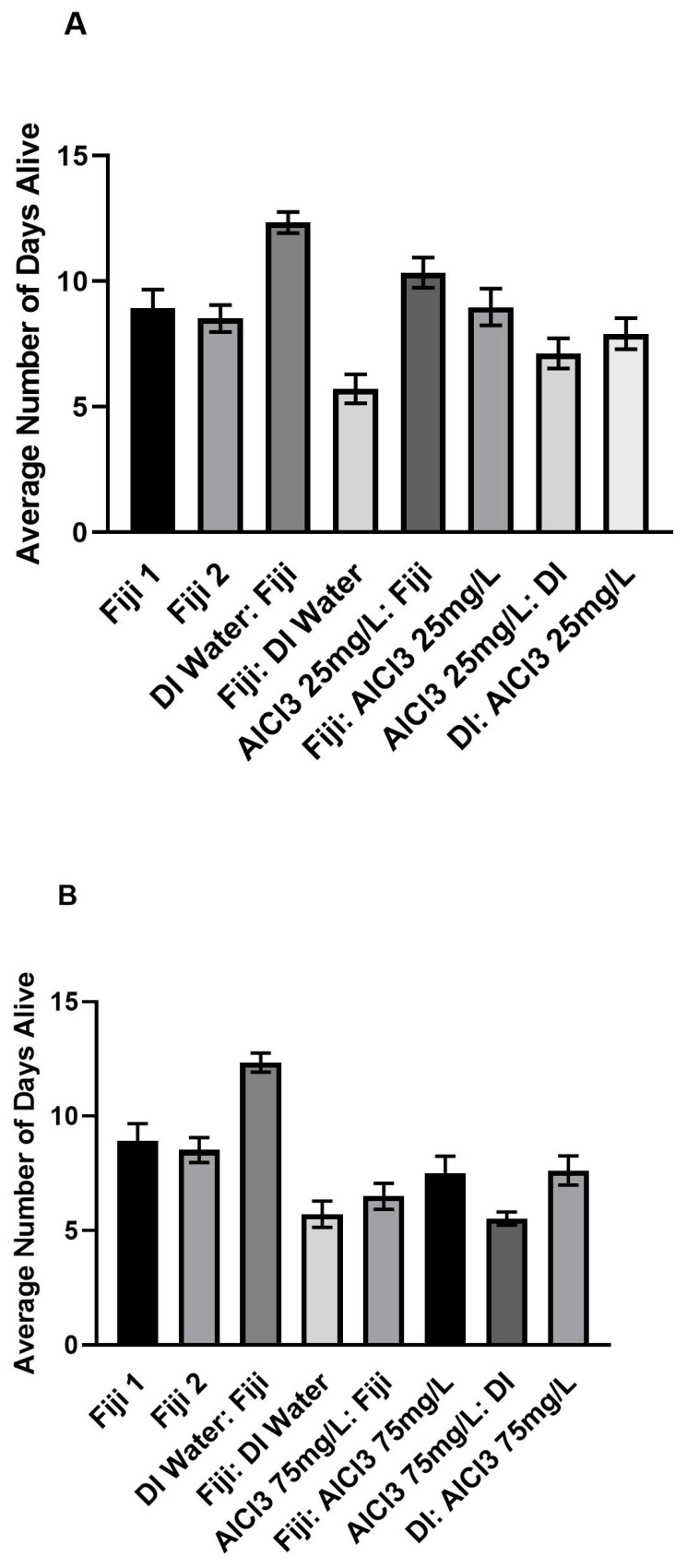

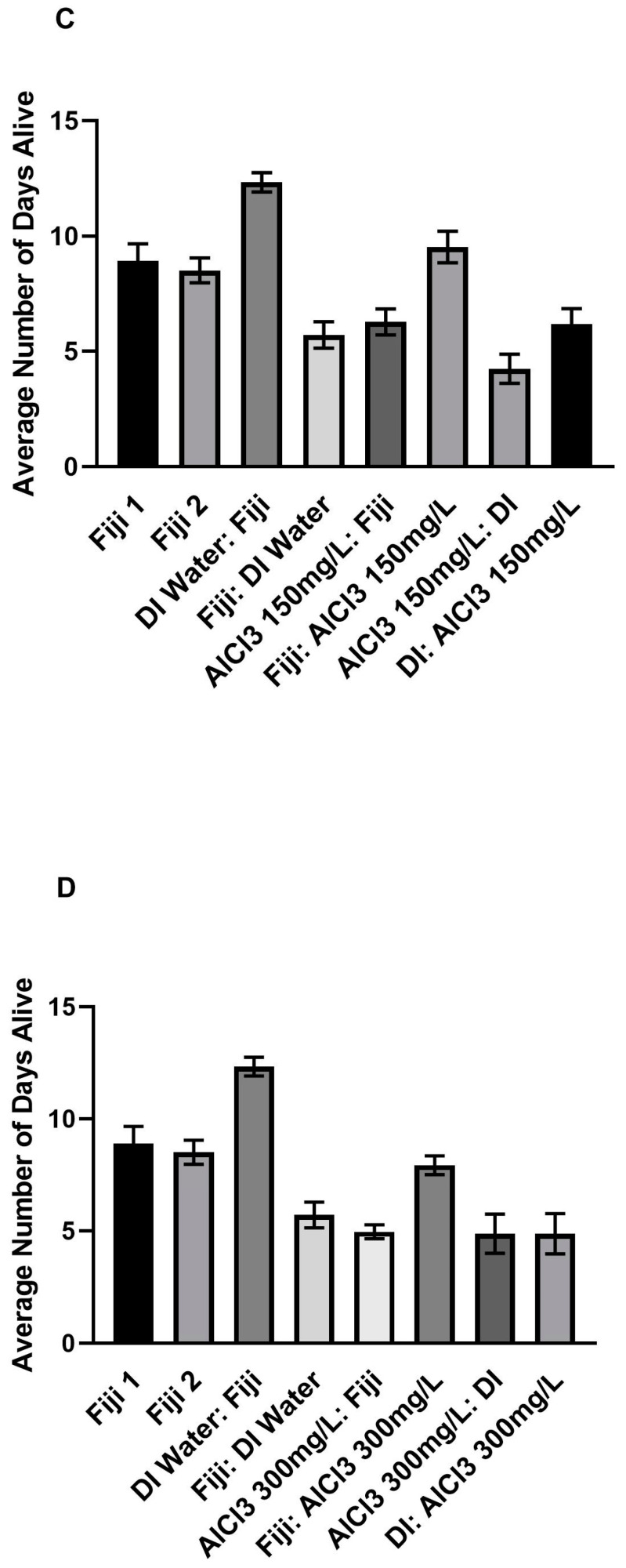
Average number of days alive by aluminum concentration with standard error of the means (SEM). The different colors represent the different groups. (**A**) Average number of days alive for AlCl3 25 mg/L and respective control group bees. (**B**) Average number of days alive for AlCl3 75 mg/L and respective control group bees. (**C**) Average number of days alive for AlCl3 150 mg/L and respective control group bees. (**D**) Average number of days alive for AlCl3 300 mg/L and respective control group bees.

The one-way ANCOVA test that compared all of the groups’ number of days alive with year as a covariate and experiment as a nested covariate was significant, *F*(21, 828) = 13.67, *p* < 0.001. Overall, the analysis showed that there were significant differences in the number of days alive between the groups. Post hoc tests revealed that on average, three of the AlCl3 groups that received Fiji water were alive significantly longer than their respective AlCl3 groups that received DI water. There was a significant main effect for experimental group, *F*(19, 828) = 11.96, *p* < 0.001. There was no significant main effect for year [*F*(1, 828) = 0.19, *p* = 0.663] or experiment cycle, *F*(1, 828) = 2.26, *p* = 0.134. Post hoc comparisons were run using a Bonferroni correction to compare the different groups, as shown in Tables S5 and S6 and Figure 3A–D and Figure 4A–D.

Only three out of all of the post hoc comparisons within the ALCL3 concentration groups were significant, as shown in Tables S5 and S6. The AlCl3 at 25 mg/L, 150 mg/L, and 300 mg/L groups each had one significant comparison. The AlCl3 at 75 mg/L was the only concentration that had no significant comparisons, as shown in Figure 3B and Figure 4B.

There was a significant difference in the number of days alive between the AlCl3 at 25 mg/L before Fiji and the AlCl3 at 25 mg/L before DI group after controlling for year and experiment cycle, as shown in Tables S5 and S6, Figure 3A and Figure 4A. The AlCl3 at 25 mg/L before Fiji group (*M* = 10.34, *SD* = 3.39) were alive for significantly more days compared to the AlCl3 at 25 mg/L before DI group (*M* = 7.13, *SD* = 3.51), *p* < 0.001. None of the other comparisons within the 25 mg/L concentration were significant, as shown in Tables S5 and S6.

There was a significant difference in the number of days alive between the AlCl3 at 150 mg/L before DI and the AlCl3 at 150 mg/L after Fiji group after controlling for year and experiment cycle, as shown in Tables S5 and S6, Figure 3C and Figure 4C. The AlCl3 at 150 mg/L before DI group (*M* = 4.25, *SD* = 3.60) were alive for significantly fewer days compared to the AlCl3 at 150 mg/L after Fiji group (*M* = 9.53, *SD* = 3.92), *p* < 0.001. None of the other comparisons within the 150 mg/L concentration were significant, as shown in Tables S5 and S6.

There was also a significant difference in the number of days alive between the AlCl3 at 300 mg/L before DI and the AlCl3 at 300 mg/L after Fiji group after controlling for year and experiment cycle, as shown in Tables S5 and S6, Figure 3D and Figure 4D. The AlCl3 at 300 mg/L before DI group (*M* = 4.88, *SD* = 1.67) were alive for significantly fewer days compared to the AlCl3 at 300 mg/L after Fiji group (*M* = 7.94, *SD* = 2.36), *p* = 0.008. None of the other comparisons within the 300 mg/L concentration were significant, as shown in Tables S5 and S6.

The results of mortality comparisons within the 16-day time period indicated that bees in the AlCl3 at 150 mg/L after DI group (*M* = 4.25, *SD* = 3.60) had the most rapid mortality followed by the AlCl3 at 300 mg/L before (*M* = 4.88, *SD* = 1.67) and after DI (*M* = 4.88, *SD* = 1.16) groups. Overall, both the AlCl3 at 300 mg/L before and after DI groups were alive for an average of 4.88 days independently, which was lower than the AlCl3 at 300 mg/L after Fiji group (*M* = 7.94, *SD* = 2.36). Interestingly, bees in the DI water before Fiji group (*M* = 12.34, *SD* = 2.36) had the highest number of days alive of the Experimental control groups. In comparison, the Fiji 1 (*M* = 8.92, *SD* = 3.67) and Fiji 2 (*M* = 8.52, *SD* = 3.05) experimental control bees had the next highest average number of days alive. Overall, when comparing within concentration there were not a lot of significant differences in the number of days alive. However, when comparing between groups, there were significant differences between the experimental controls as well as different concentrations.

## 4. Discussion

The average daily activity and rhythmicity results from our study show some differences within the various aluminum experimental groups compared to their respective aluminum (AlCl3) and DI water groups. The AlCl3 after Fiji groups for all of the concentrations had significantly higher rhythmicity rates compared to their respective AlCl3 after DI water groups. However, only the AlCl3 at 25 mg/L, 75 mg/L, and 150 mg/L after Fiji groups had significantly higher average daily activity rates compared to their respective AlCl3 after DI water groups. This suggests that the Fiji water may have had a protective effect against the aluminum.

Additionally, for all of the concentrations, the AlCl3 before Fiji groups had significantly higher rhythmicity rates compared to their respective AlCl3 after DI water groups. Only the AlCl3 at 75 mg/L and 150 mg/L before Fiji groups had significantly higher average daily activity rates compared to their respective AlCl3 after DI water groups. In comparison, except for the AlCl3 at 25 mg/L concentration, all of the AlCl3 before DI water groups had significantly lower average daily activity rates compared to their respective AlCl3 after Fiji groups. However, all of the AlCl3 before DI water groups had significantly lower rhythmicity rates compared to their respective AlCl3 after Fiji water groups. The differences between the groups may be explained by the activity throughout the day being spread out between the different hours for the experimental groups with the activity being less spread out for the AlCl3 before and after DI water bees (Figure 1A–D).

The average daily activity and rhythmicity rates for the experimental groups were significantly different from the controls for the majority of the concentrations. The average daily activity of the AlCl3 at 25 mg/L before DI water group was the only AlCl3 group that exhibited no significant difference when compared to the zero-baseline control. Additionally, all of the other AlCl3 before and after DI water groups had significantly lower average daily activity levels compared to the zero-baseline control. In comparison, all of the AlCl3 before and after Fiji water groups had significantly higher average daily activity levels compared to the zero-baseline control. These findings could be suggestive of a prophylactic effect if silica is consumed prior to aluminum exposure. The results are of particular interest since they contrast with the results of previous experiments conducted by Chicas-Mosier et al. [17]. In the prior experiments, honey bees experienced opposing effects of rhythmicity and activity with bees exhibiting hyperactivity at the lower aluminum exposure doses compared to controls [17]. The majority of the bees in our experiment that received aluminum before or after DI water exhibited significant decreases in both average daily activity and rhythmicity regardless of the concentration they ingested. These differences may be due to seasonality or year since our tests indicated that both year and experiment cycle impacted rhythmicity.

The majority of the bees in the groups that received AlCl3 paired with Fiji water with the exception of the AlCl3 at 300 mg/L before Fiji group had significantly higher average daily activity levels compared to the zero-baseline, as shown in Figure 2A–D. Researchers have determined that aluminum can cause hyperactivity, thus we would expect to observe higher levels of activity [15,16]. Specifically, at lower concentrations, the disruption to the cholinergic system can occur, which can result in hyperactivity [17]. Chicas-Mosier et al. [17] determined that bees exposed to 10.4, 25, and 75 mg/L of aluminum exhibited higher levels of average daily activity compared to the control bees.

Interestingly, the majority of the AlCl3 before and after DI water groups with the exception of the AlCl3 25 mg/L→DI group had significantly lower average daily activity levels compared to the zero-baseline. These bees exhibited decreases in activity during the daytime and increases during the nighttime as seen in Figure 1A–D. The lower average activity in these groups might be due to exhaustion, leading to lower activity [17]. The cholinergic system can be over-activated when exposed to high concentrations of aluminum, which results in later physical exhaustion [17]. This exhaustion is exhibited in lower activity and rhythmicity levels. The bees that received aluminum before and after Fiji water may not have exhibited these effects of aluminum exposure due to protective effects from the AlCl3.

For the AlCl3 at 300 mg/L concentration groups, these results might be the effect of a similar finding where the 300 mg/L exposure spiked AChE tissue activity compared to other doses (more AChE → less ACh → less movement) [9]. In comparison, the Fiji 1 group had a significant spike in average rhythmicity levels around 6:00 AM−6:00 PM compared to the other groups who exhibited decreases or no change during that time (Figure 1A–D). The implications of the heightened movement in the middle of the night by the other groups could contribute to timing issues that cause them to miss the flowering schedule, exhaustion during normal active hours and plant phenology (the study of annual plant development such as flowering schedule). Timing plays an important role in honey bee foraging due to plant phenology. It is important for bees to forage on plants during the flowering phase to gather the most resources [31].

Additionally, we observed significant differences between the Fiji 1 and Fiji 2 bees for rhythmicity and average daily activity from the zero-baseline. Fiji water contains sodium and other micronutrients including calcium and magnesium [32]. This may account for some of the differences that we observe in the Fiji water-only bees’ rhythmicity and average daily activity. Researchers determined that bees prefer solutions with sodium in them [33]. Differences between the Fiji water 1 and Fiji water 2 groups may also be due to yearly differences since the data collection for the Fiji 1 bees was conducted in 2020 and the data collection for the Fiji 2 bees was conducted in 2022.

There are several factors that may account for the differences discovered in these experiments. The honey bees used for this experiment were all collected from laboratory hives in Oklahoma, which consisted of *Apis mellifera*. The findings might be different depending on the specific subspecies or region of the tested honey bees, as has been suggested in prior publications [17]. For example, *A. mellifera mellifera* has been shown to exhibit less of a change in circadian rhythmicity following exposure to aluminum compared to other subspecies (*A. mellifera carnica* & *A. mellifera caucasica*) [17].

We combined our data after controlling for variation by subtracting the respective baseline control values from each group. These differences between control groups could be due to seasonal variation which may be an important factor to consider when conducting future studies. It is important to note that the data was collected during two different years, which also may account for some of the differences.

Previous research has determined that seasonal variation likely affects honey bee behavior [31,34]. Scheiner et al. [34] discovered that pollen and non-pollen foragers exhibited variations in their responsiveness to sucrose during their four-week study. The competitive pressure for foraging nectar has been determined to vary with the season, which can alter honey bee foraging behavior [31].

Our survival curve indicated that a majority of the bees were dead by the end of 14 days of treatment. This high mortality rate may be due to several factors such as the use of DI water and a decrease in survival over the years in captive studies. In Chicas-Mosier et al. [17], the researchers observed honey bee survival up to approximately 22 days. In comparison, Delkash-Roudsari et al. [29] used a 14-day dosing period or until all bees were dead for data collection, which is lower than the 16-day period we used. Like other experiments, we used DI water in our experiment [9,17,18,29,35]. The bees in both our control and experimental solution groups consumed DI water. DI water has also been used as a control with honey bee brood [36]. We recognize that this the use of DI water for a control is non-standard. Since the DI water was used to make both experimental solutions as well as for the DI water control, we believe that the control should offer a direct comparison to the experimental groups even if it is not ideal. Researchers have obtained evidence that honey bees have seasonal preferences towards micronutrients [37]. Specifically, they had strong preferences for salt compared to DI water during the summer and winter seasons [37].

Research on the median lifespans for control groups in cage studies indicated that survival had drastically decreased since 1970 with a median survival of 10 days for control groups from cage studies in 2019 [33]. The researchers determined that caged bees provided with tap water (67%) had a higher probability of surviving 21 days compared to bees that were offered DI water alone or with 1% sodium chloride (NaCl) (~37.5%) [33]. As a result, it is important to note that the use of the DI water could contribute to the higher mortality rates that we observed across groups. However, since both controls and experimental groups consumed DI water, we suggest that our findings provide a beneficial comparison between the groups.

Using a different form of silica might also result in different findings. Researchers obtained evidence that oligomeric but not monomeric silica impeded the absorption of aluminum in the digestive tract [24]. Aqueous silica can be found naturally in fresh water with concentrations ranging from 1 to 100 mg/L [38]. One of Fiji water’s properties is that it is high in silica with concentrations of 85 mg/L due to the silica leaching into the water from the volcanic rock [38,39]. As a result, using a different form of silica may influence the absorption of the aluminum. In addition, providing the silica either before or after the aluminum but not switching between the two every four days might also provide different results.

Future research should examine the possible role of different silicate compounds at various concentrations to determine the impact on the absorption of aluminum. Previous research shows that bees are affected by doses of aluminum that match bioavailable concentrations of the metal and are bringing it back to their hive [17,18,19,20]. It is crucial to minimize the effect of aluminum to improve hive health and increase honey bee longevity.

Honey bees play an integral part in agricultural production in the United States economy [1,3]. Aluminum is increasing in bioavailability through acidification from carbon emissions and mining practices, which may lead to a higher risk of honey bees being exposed to this metal. Aluminum toxicity has negative impacts on honey bee behavior and physiology, making it difficult for them to perform crucial survival behaviors such as foraging [9,17,18]. As a result, it is important for researchers to develop methods that help to minimize the effects of aluminum on honey bees. Honey bees are an important part of the economy and help maintain the stability of the worldwide food supply. Exposure to aluminum has resulted in detrimental effects on honey bee foraging and mortality. As such, it is important to investigate mechanisms that may decrease the effects of aluminum on honey bees. The results from these experiments suggest that further study is needed to determine whether silica can possibly be used as a preventative measure against aluminum toxicity.

## 5. Conclusions

Future studies should focus on possible preventative measures that could be used by commercial beekeepers as well as the general public. Finding a prevention measure that is inexpensive and readily available, such as silicated water, will make it easier for beekeepers and the general public to sustain use of this method. Researchers have only started to determine possible measures for minimizing aluminum uptake in honey bees. This type of research will have wide-ranging impact on commercial and hobby honey beekeepers as well as the general public.

## Figures and Tables

**Table 1:** Bee Exposure Groups and Formulas.

Treatment	Aluminum Solution	Days per Treatment	Solute Added
DI Water (control)	AlCl3 0 mg/L	16	NA
Fiji Water (Experimental Control)	AlCl3 0 mg/L	16	NA
DI water→Fiji (Experimental Design Control)	AlCl3 0 mg/L	4 DI→4 Fiji→4 DI→4 Fiji	NA
Fiji→DI water (Counterbalanced Experimental Design Control)	AlCl3 0 mg/L	4 Fiji→4 DI→4 Fiji→4 DI	NA
AlCl3 first	AlCl3 25 mg/L	4 AlCl3→4 Fiji→4 AlCl3→4 Fiji	125 mg for 1-L DI
AlCl3 first	AlCl3 75 mg/L	4 AlCl3→4 Fiji→4 AlCl3→4 Fiji	375 mg for 1-L DI
AlCl3 first	AlCl3 150 mg/L	4 AlCl3→4 Fiji→4 AlCl3→4 Fiji	750 mg for 1-L DI
AlCl3 first	AlCl3 300 mg/L	4 AlCl3→4 Fiji→4 AlCl3→4 Fiji	1500 mg for 1-L DI
Fiji water first	AlCl3 25 mg/L	4 Fiji→4 AlCl3→4 Fiji→4 AlCl3	125 mg for 1-L DI
Fiji water first	AlCl3 75 mg/L	4 Fiji→4 AlCl3→4 Fiji→4 AlCl3	375 mg for 1-L DI
Fiji water first	AlCl3 150 mg/L	4 Fiji→ 4 AlCl3→4 Fiji→4 AlCl3	750 mg for 1-L DI
AlCl3 first	AlCl3 25 mg/L	4 AlCl3→4 DI→4 AlCl3→4 DI	125 mg for 1-L DI
AlCl3 first	AlCl3 75 mg/L	4 AlCl3→4 DI→4 AlCl3→4 DI	375 mg for 1-L DI
AlCl3 first	AlCl3 150 mg/L	4 AlCl3→4 DI→4 AlCl3→4 DI	750 mg for 1-L DI
AlCl3 first	AlCl3 300 mg/L	4 AlCl3→4 DI→4 AlCl3→4 DI	1500 mg for 1-L DI
DI water first	AlCl3 25 mg/L	4 DI→4 AlCl3→4 DI→4 AlCl3	125 mg for 1-L DI
DI water first	AlCl3 75 mg/L	4 DI→4 AlCl3→4 DI→4 AlCl3	375 mg for 1-L DI
DI water first	AlCl3 150 mg/L	4 DI→ 4 AlCl3→4 DI→4 AlCl3	750 mg for 1-L DI
DI water first	AlCl3 300 mg/L	4 DI→4 AlCl3→4 DI→4 AlCl3	1500 mg for 1-L DI

Note. The → indicates the switch between solutions.

## Data Availability

Data will be available online.

## Supplementary Materials

The following supporting information can be downloaded at: https://www.mdpi.com/article/10.3390/insects14020211/s1, Table S1: Pairwise comparisons with a Bonferroni correction for the ANCOVA utilizing the rhythmicity data. Effects were compared to a zero-baseline using on-sample *t*-tests; Table S2: Significance table for experimental group rhythmicity data. Effects are compared to a zero-baseline; Table S3: Significance table for one-sample *t*-tests comparing average daily activity to a zero-baseline for aluminum groupings; Table S4: Pairwise comparisons with a Bonferroni correction for the average daily activity ANCOVA by aluminum concentration; Table S5: Combined mortality data ANCOVA comparisons; Table S6: Pairwise comparisons with a Bonferroni correction for the ANCOVA for the number of days alive by aluminum concentration; Figure S1: Monitor apparatus with pipes; Figure S2: Side view of monitor apparatus with tube to show centerline.
