# Supplementary material for: Preliminary Evidence That Fiji Water Has Protective Effects against Aluminum Toxicity in Honey Bees (Apis mellifera)
Source: Insects. 2023 Feb 20;14(2):211. doi: 10.3390/insects14020211 (PMC9958646; doi:10.3390/insects14020211)
Supplement: Supplementary file 1 [file insects-14-00211-s001.zip › insects-2111861-Supplementals.pdf]

Table S1. Pairwise comparisons with a Bonferroni correction for the ANCOVA utilizing the rhythmicity data by aluminum grouping.

Treatment (mg/L) Pair Group 1	Group 2	Significance between groups	Group 1 comparison to Group 2
		**AlCl3 25mg/L groups**	
AlCl3 25→Fiji	AlCl3 25→DI	***	Higher
AlCl3 25→Fiji	Fiji→AlCl3 25	1.000	
AlCl3 25→Fiji	DI→AlCl3 25	***	Higher
AlCl3 25→DI	Fiji→AlCl3 25	***	Lower
AlCl3 25→DI	DI→AlCl3 25	***	Higher
Fiji→AlCl3 25	DI→AlCl3 25	***	Higher
		**AlCl3 75mg/L groups**	
AlCl3 75→Fiji	AlCl3 75→DI	***	Higher
AlCl3 75→Fiji	Fiji→AlCl3 75	1.000	
AlCl3 75→Fiji	DI→AlCl3 75	***	Higher
AlCl3 75→DI	Fiji→AlCl3 75	***	Lower
AlCl3 75→DI	DI→AlCl3 75	***	Lower
Fiji→AlCl3 75	DI→AlCl3 75	***	Higher
		**AlCl3 150mg/L groups**	
AlCl3 150→Fiji	AlCl3 150→DI	***	Higher
AlCl3 150→Fiji	Fiji→AlCl3 150	***	Lower
AlCl3 150→Fiji	DI→AlCl3 150	***	Higher
AlCl3 150→DI	Fiji→AlCl3 150	***	Lower
AlCl3 150→DI	DI→AlCl3 150	***	Lower
Fiji→AlCl3 150	DI→AlCl3 150	***	Higher
		**AlCl3 300mg/L groups**	
AlCl3 300→Fiji	AlCl3 300→DI	***	Higher
AlCl3 300→Fiji	Fiji→AlCl3 300	***	Lower
AlCl3 300→Fiji	DI→AlCl3 300	***	Higher
AlCl3 300→DI	Fiji→AlCl3 300	***	Lower
AlCl3 300→DI	DI→AlCl3 300	.020*	Lower
Fiji→AlCl3 300	DI→AlCl3 300	***	Higher

Note. The means and standard deviations can be found in Table S2. Comparison with Group 2 information is only shown for groups with significant p values when compared to a control. Each group had a sample size of $n = 32$, so the combined sample size for each treatment pair was $n = 62$. * $p < 0.05$, ** $p < 0.001$, and *** $p < 0.0001$

Table S2. Significance table for experimental group rhythmicity data. Effects were compared to a zero baseline using one-sample *t*-tests.

Treatment		mg/L	M	Sd	df	*t*-value	*p*-value	Effect
Fiji water 1	Day	0	35.89	82.34	263	7.082	***	Higher
Fiji water 2		0	-190.38	778.39	351	-4.589	***	Lower
DI Water→Fiji		0	34.00	88.51	351	7.207	***	Higher
Fiji→DI water		0	-23.72	69.78	351	-6.377	***	Lower
AlCl3→Fiji		25	-49.49	53.38	351	-17.394	***	Lower
AlCl3→Fiji		75	-.60	52.17	351	-0.214	0.830	
AlCl3→Fiji		150	10.38	68.04	351	2.863	0.004**	Higher
AlCl3→Fiji		300	-91.52	34.45	351	-49.847	***	Lower
Fiji→AlCl3		25	-38.37	92.38	351	-7.792	***	Lower
Fiji→AlCl3		75	-10.77	46.33	351	-4.360	***	Lower
Fiji→AlCl3		150	16.75	91.72	351	3.427	0.001**	Higher
Fiji→AlCl3		300	-17.46	49.34	351	-6.640	***	Lower
AlCl3→DI water		25	44.18	1052.34	351	0.788	0.431	
AlCl3→DI water		75	-1142.82	437.59	351	-49.00	***	Lower
AlCl3→DI water		150	-363.66	371.08	351	-18.386	***	Lower
AlCl3→DI water		300	-560.97	532.87	351	-19.751	***	Lower
DI water→ AlCl3		25	-685.60	699.60	351	-18.386	***	Lower
DI water→ AlCl3		75	-541.17	738.59	351	-13.747	***	Lower
DI water→ AlCl3		150	-157.43	456.14	351	-6.475	***	Lower
DI water→ AlCl3		300	-433.44	611.41	351	-13.301	***	Lower
Fiji water 1	Night	0	11.99	51.66	312	4.105	***	Higher
Fiji water 2		0	-112.26	375.02	415	-6.105	***	Lower
DI Water→Fiji		0	19.91	63.90	416	6.32	***	Higher
Fiji→DI water		0	-9.52	31.81	416	-6.108	***	Lower
AlCl3→Fiji		25	-14.67	37.19	416	-8.053	***	Lower
AlCl3→Fiji		75	1.60	39.94	416	0.817	0.415	
AlCl3→Fiji		150	4.50	39.72	416	2.312	0.021*	Higher
AlCl3→Fiji		300	-35.84	23.93	416	-30.585	***	Lower
Fiji→AlCl3		25	-18.34	42.03	416	-8.910	***	Lower
Fiji→AlCl3		75	-12.22	25.87	416	-9.647	***	Lower
Fiji→AlCl3		150	6.09	47.82	416	2.600	0.010*	Higher
Fiji→AlCl3		300	-6.68	32.70	416	-4.173	***	Lower
AlCl3→DI water		25	-23.36	522.61	415	-0.911	0.363	
AlCl3→DI water		75	-469.68	288.11	415	-33.250	***	Lower
AlCl3→DI water		150	-112.79	196.66	415	-11.698	***	Lower
AlCl3→DI water		300	-285.77	182.21	415	-31.988	***	Lower
DI water→ AlCl3		25	-324.66	384.15	415	-17.238	***	Lower
DI water→ AlCl3		75	-347.97	478.97	415	-14.817	***	Lower
DI water→ AlCl3		150	-9.98	334.37	415	-0.609	0.543	

Table S2. (continued).

**DI water→ AlCl3**		**300**	**-238.04**	**254.68**	**415**	**-19.063**	*******	**Lower**
Fiji water 1	Avg. 24hr Activity	0	22.92	68.43	576	8.046	**	Higher
Fiji water 2		0	-148.06	595.72	767	-6.888	**	Lower
DI Water→Fiji		0	26.36	76.43	768	9.564	**	Higher
Fiji→DI water		0	-16.02	53.14	768	-8.359	**	Higher
AlCl3→Fiji		25	-30.61	48.51	768	-17.497	**	Lower
AlCl3→Fiji		75	0.59	45.93	768	0.358	0.720	
AlCl3→Fiji		150	7.19	54.58	768	3.654	**	Higher
AlCl3→Fiji		300	-61.33	40.28	768	-42.217	**	Lower
Fiji→AlCl3		25	-27.51	70.41	768	-10.834	**	Lower
Fiji→AlCl3		75	-11.56	36.66	768	-8.741	**	Lower
Fiji→AlCl3		150	10.97	71.49	768	4.255	**	Higher
Fiji→AlCl3		300	-11.62	41.48	768	-7.766	**	Higher
AlCl3→DI water		25	7.61	809.75	767	0.260	0.795	
AlCl3→DI water		75	-778.20	495.16	767	-43.554	***	Lower
AlCl3→DI water		150	-227.77	315.57	767	-20.002	***	Lower
AlCl3→DI water		300	-411.91	408.33	767	-27.956	***	Lower
DI water→ AlCl3		25	-490.09	579.84	767	-23.423	***	Lower
DI water→ AlCl3		75	-436.52	618.91	767	-19.546	***	Lower
DI water→ AlCl3		150	-77.56	401.39	767	-5.355	***	Lower
DI water→ AlCl3		300	-327.60	464.39	767	-19.550	***	Lower

Note: Combined DI (n = 218): Day (M = 569.42, SD = 843.32), Night (M = 225.68, SD = 414.72), Avg. 24 hr Activity (M = 383.12, SD = 669.48). The n for all AlCl3 before and after DI, AlCl3 before and after Fiji water, and Fiji 2 is 32. The n for Fiji water 2 is 24. * $p < 0.05$, ** $p < 0.01$, and *** $p < 0.001$

Table S3. Significance table for one-sample *t*-tests comparing aluminum groupings average daily activity to a zero baseline.

Treatment (mg/L)	N data points	M	SD	*t*	df	*p*	Lower 95% CI	Upper 95% CI
Fiji water 1	214	2394.35	3324.58	10.540	213	***	1946.38	2842.32
Fiji water 2	960	-109.16	832.27	-4.064	959	***	-161.87	-56.45
DI Water→Fiji	395	1715.21	3229.45	10.556	394	***	1395.75	2034.67
Fiji→DI water	184	1504.34	2629.70	7.760	183	***	1121.85	1886.84
AlCl3 25→Fiji	331	643.08	2212.24	5.289	330	***	403.88	882.28
AlCl3 75→Fiji	208	1532.74	2707.88	8.163	207	***	1162.58	1902.91
AlCl3 150→Fiji	200	1958.36	3068.83	9.025	199	***	1530.44	2386.27
AlCl3 300→Fiji	157	248.77	1550.81	2.010	156	.046*	4.289	493.24
Fiji→AlCl3 25	287	1022.76	2546.14	6.805	268	***	726.93	1318.58
Fiji→AlCl 3 75	255	801.10	2051.49	6.236	254	***	548.10	1054.10
Fiji→AlCl3 150	305	1592.61	3151.64	8.825	304	***	1237.49	1947.72
Fiji→AlCl3 300	254	752.48	2037.25	5.887	253	***	500.74	1004.23
AlCl3 25: DI	799	18.34	1116.22	0.464	798	0.642	-59.17	95.86
AlCl3 75: DI	768	-718.89	740.99	-26.886	767	***	-771.38	-666.40
AlCl3 150: DI	576	-338.41	813.07	-9.99	575	***	-404.95	-271.87
AlCl3 300: DI	576	-595.04	809.19	-17.648	575	***	-661.26	-528.82
DI: AlCl3 25	864	-443.90	921.70	-14.156	863	***	-505.44	-382.35
DI: AlCl3 75	1024	-872.82	3402.04	-8.210	1023	***	-1081.45	-664.21
DI: AlCl3 150	832	-214.79	791.79	-7.825	831	***	-268.67	-160.91
DI: AlCl3 300	832	-371.71	784.12	-13.674	831	***	-425.07	-318.36

Note: N=1 monitors for each concentration. N data points is the number of crossings. The DI control group (N = 218) had 9 monitors (one for each data collection period) and are not included due to the zero baseline representing the controls. The N for all AlCl3 before and after DI, AlCl3 before and after Fiji water, and Fiji 2 is 32. * $p < 0.05$, ** $p < 0.01$, *** $p < 0.001$.

Table S4. Pairwise comparisons with a Bonferroni correction for the average daily activity ANCOVA by aluminum concentration.

Treatment(mg/L) Pair Group 1	Group 2	Significance between groups	Group 1 comparison to Group 2
**AlCl3 25mg/L groups**			
AlCl3 25→Fiji	AlCl3 25→DI	1.000	
AlCl3 25→Fiji	Fiji→AlCl3 25	1.000	
AlCl3 25→Fiji	DI→AlCl3 25	0.120	
AlCl3 25→DI	Fiji→AlCl3 25	0.922	
AlCl3 25→DI	DI→AlCl3 25	0.005*	Higher
Fiji→AlCl3 25	DI→AlCl3 25	****	Higher
**AlCl3 75mg/L groups**			
AlCl3 75→Fiji	AlCl3 75→DI	****	Higher
AlCl3 75→Fiji	Fiji→AlCl3 75	****	Higher
AlCl3 75→Fiji	DI→AlCl3 75	****	Higher
AlCl3 75→DI	Fiji→AlCl3 75	****	Lower
AlCl3 75→DI	DI→AlCl3 75	.1.00	
Fiji→AlCl3 75	DI→AlCl3 75	****	Higher
**AlCl3 150mg/L groups**			
AlCl3 150→Fiji	AlCl3 150→DI	****	Higher
AlCl3 150→Fiji	Fiji→AlCl3 150	1.00	
AlCl3 150→Fiji	DI→AlCl3 150	****	Higher
AlCl3 150→DI	Fiji→AlCl3 150	****	Lower
AlCl3 150→DI	DI→AlCl3 150	1.00	
Fiji→AlCl3 150	DI→AlCl3 150	****	Higher
**AlCl3 300mg/L groups**			
AlCl3 300→Fiji	AlCl3 300→DI	1.000	
AlCl3 300→Fiji	Fiji→AlCl3 300	1.00	
AlCl3 300→Fiji	DI→AlCl3 300	1.00	
AlCl3 300→DI	Fiji→AlCl3 300	****	Lower
AlCl3 300→DI	DI→AlCl3 300	1.000	
Fiji→AlCl3 300	DI→AlCl3 300	0.080	

Note: Group 1 and Group 2 are compared against each other in the respective analysis. Comparison with Group 2 information is only shown for groups with significant *p* values when compared to Group 2. The n for all AlCl3 before and after DI, AlCl3 before and after Fiji water, and Fiji 2 is 32. * $p < 0.05$, ** $p < 0.01$, *** $p < 0.001$, and **** $p < 0.0001$.

Table S5. Combined Mortality Data ANCOVA comparisons.

**Treatment (mg/L)**	***n***	***M***	***SD***	***SE***	***p***	***Average Numbers of Days Alive Compared with respective control***
Fiji water 1	24	8.92	3.67	0.75	.010*	Higher
Fiji water 2	32	8.52	3.05	0.54	.107	
DI Water: Fiji	32	12.34	2.36	0.42	***	Higher
Fiji: DI water	32	5.72	3.30	0.58	***	Lower
AlCl3(25): Fiji	32	10.34	3.39	0.60	.996	
AlCl3(75): Fiji	32	6.50	3.20	0.57	.959	
AlCl3(150): Fiji	32	6.28	3.18	0.56	.193	
AlCl3(300): Fiji	32	4.97	1.77	0.31	***	Higher
Fiji: AlCl3(25)	32	8.97	4.10	0.73	.960	
Fiji: AlCl3(75)	32	7.50	4.32	0.76	.418	
Fiji: AlCl3(150)	32	9.53	3.92	0.69	.945	
Fiji: AlCl3(300)	32	7.94	2.36	0.42	.021*	Lower
AlCl3(25): DI	32	7.13	3.51	0.60	.004*	Lower
AlCl3(75): DI	32	5.53	3.41	0.29	.001*	Lower
AlCl3(150): DI	32	4.25	3.60	0.630	.002*	Lower
AlCl3(300): DI	32	4.88	1.67	0.88	***	Lower
DI: AlCl3(25)	32	7.91	2.01	0.62	.357	
DI: AlCl3(75)+	32	7.63	1.50	0.64	***	Lower
DI: AlCl3(150)	32	6.19	1.59	0.67	.870	
DI: AlCl3(300)	32	4.88	1.16	0.90	.014*	Lower

Note. Comparison is shown based on mean number of days alive. An independent samples *t*-test was run for one group as indicated by the + due to the number of groups. The n for all AlCl3 before and after DI, AlCl3 before and after Fiji water, and Fiji 2 is 32. * $p < 0.05$, ** $p < 0.01$, *** $p < 0.001$.

Table S6. Pairwise comparisons with a Bonferroni correction for the ANCOVA for the number of days alive by aluminum concentration.

Treatment(mg/L) Pair Group 1	Group 2	Significance between groups	Group 1 comparison to Group 2
	**ALCL3 25mg/L groups**		
AlCl3 25→Fiji	AlCl3 25→DI	0.001**	Higher
AlCl3 25→Fiji	Fiji→AlCl3 25	1.00	
AlCl3 25→Fiji	DI→AlCl3 25	0.421	
AlCl3 25→DI	Fiji→AlCl3 25	0.284	
AlCl3 25→DI	DI→AlCl3 25	1.000	
Fiji→AlCl3 25	DI→AlCl3 25	1.000	
	**ALCL3 75mg/L groups**		
AlCl3 75→Fiji	AlCl3 75→DI	1.000	
AlCl3 75→Fiji	Fiji→AlCl3 75	1.000	
AlCl3 75→Fiji	DI→AlCl3 75	1.000	
AlCl3 75→DI	Fiji→AlCl3 75	0.698	
AlCl3 75→DI	DI→AlCl3 75	1.000	
Fiji→AlCl3 75	DI→AlCl3 75	1.000	
	**ALCL3 150mg/L groups**		
AlCl3 150→Fiji	AlCl3 150→DI	0.234	
AlCl3 150→Fiji	Fiji→AlCl3 150	0.324	
AlCl3 150→Fiji	DI→AlCl3 150	1.00	
AlCl3 150→DI	Fiji→AlCl3 150	****	Lower
AlCl3 150→DI	DI→AlCl3 150	1.000	
Fiji→AlCl3 150	DI→AlCl3 150	0.011*	Higher
	**ALCL3 300mg/L groups**		
AlCl3 300→Fiji	AlCl3 300→DI	1.000	
AlCl3 300→Fiji	Fiji→AlCl3 300	0.192	
AlCl3 300→Fiji	DI→AlCl3 300	1.000	
AlCl3 300→DI	Fiji→AlCl3 300	0.008**	Lower
AlCl3 300→DI	DI→AlCl3 300	1.000	
Fiji→AlCl3 300	DI→AlCl3 300	1.000	

Note: Group 1 and Group 2 are compared against each other in the respective analysis. Comparison with Group 2 information is only shown for groups with significant p values when compared to Group 2. The n for all groups is 32 bees. * $p < 0.05$, ** $p < 0.01$, *** $p < 0.001$, and **** $p < 0.0001$

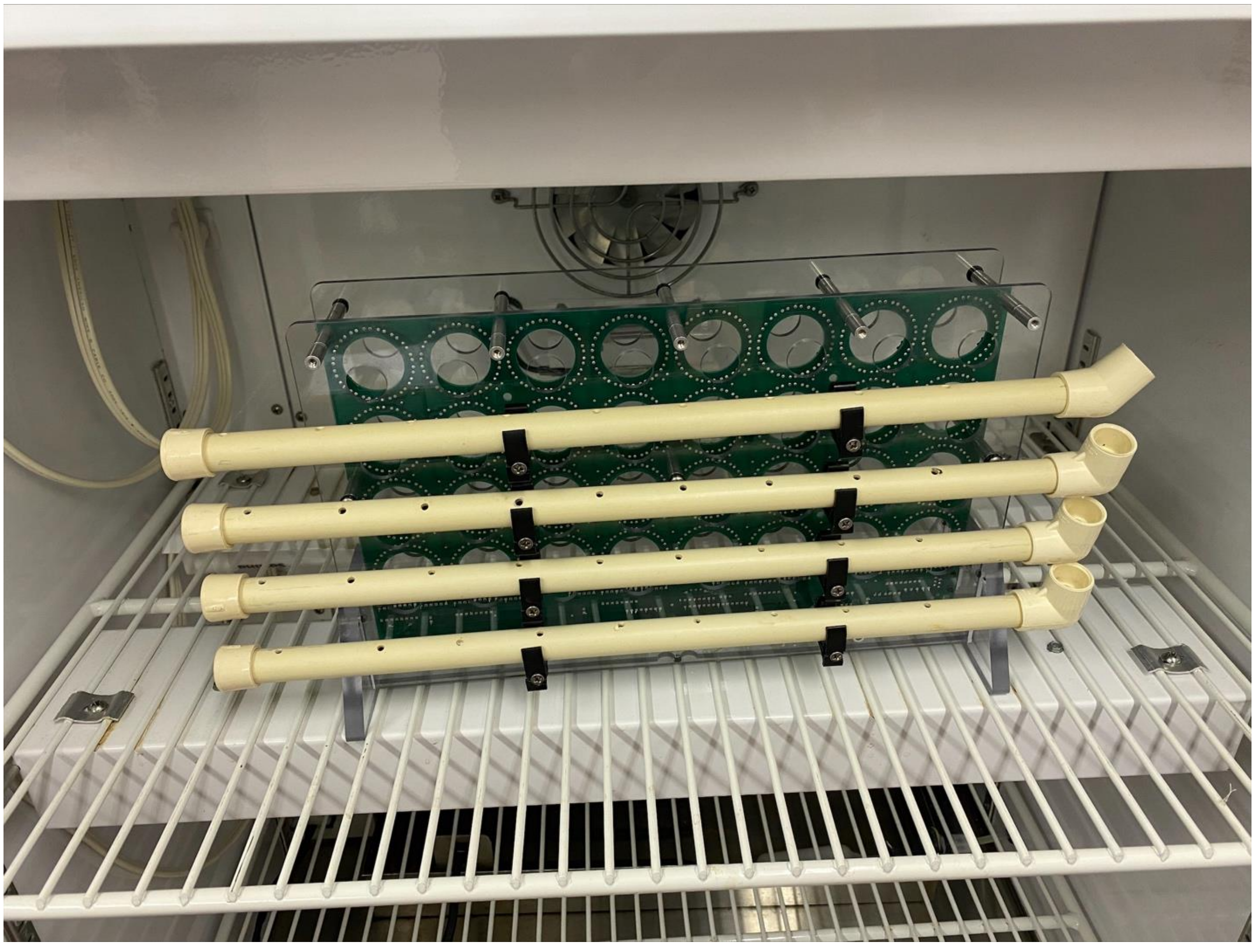

Figure S1: Monitor apparatus with pipes

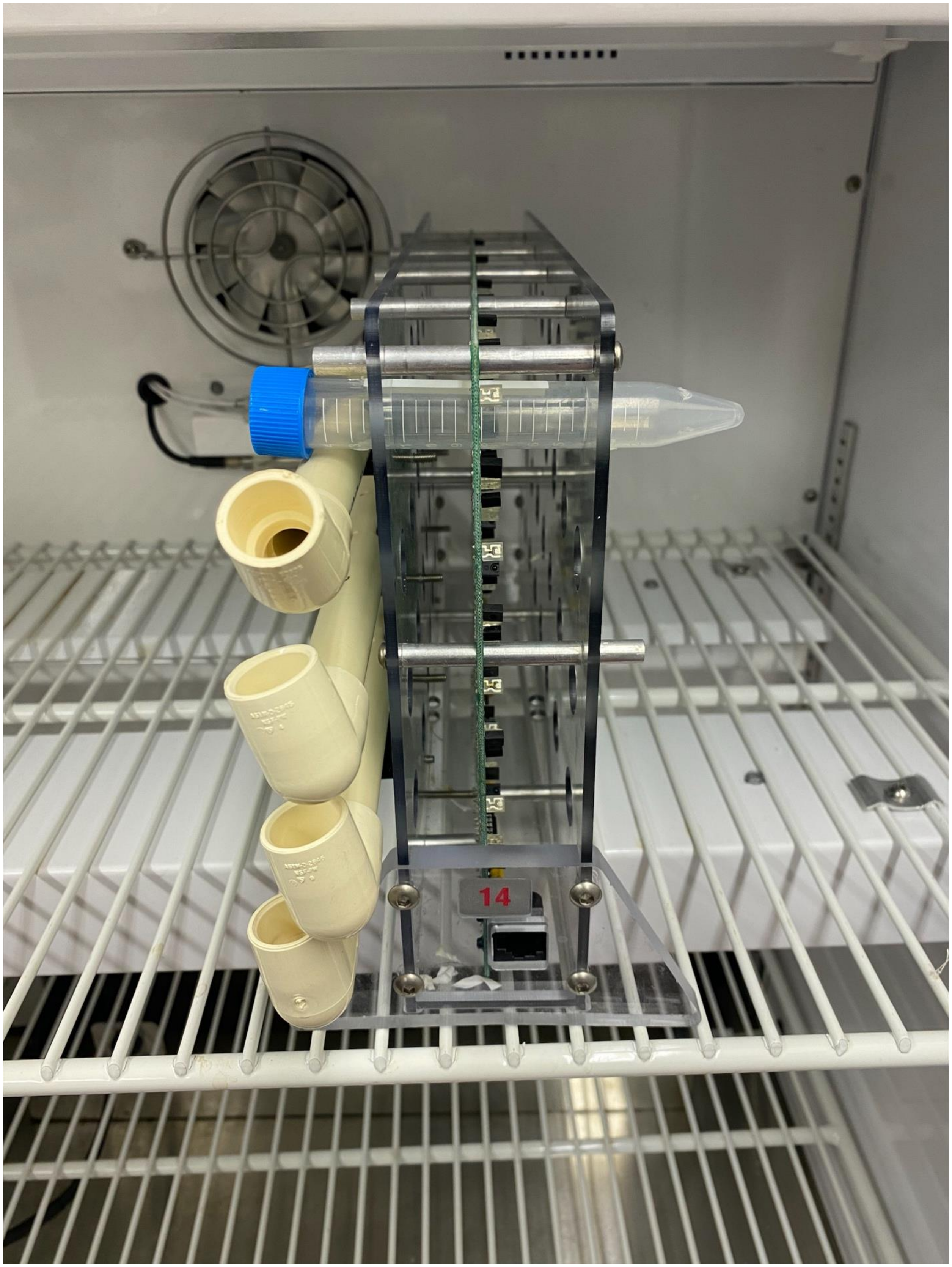

Figure S2: Side view of monitor apparatus with tube to show centerline.
